# New strategy for cancer immunotherapy: using live engineered bacteria for metabolic modulation

**DOI:** 10.1038/s41392-021-00829-4

**Published:** 2021-12-06

**Authors:** Tong Li, Wenjing Wang

**Affiliations:** 1 State Key Laboratory of Biotherapy, Cancer Center, West China Hospital, Sichuan University and Collaborative Innovation Center for Biotherapy, Chengdu, 610041 China; 2 Laboratory of Human Diseases and Immunotherapies, West China Hospital, Sichuan University, Chengdu, 610041 China

**Keywords:** Cancer metabolism, Tumour immunology

**Fig. 1:**
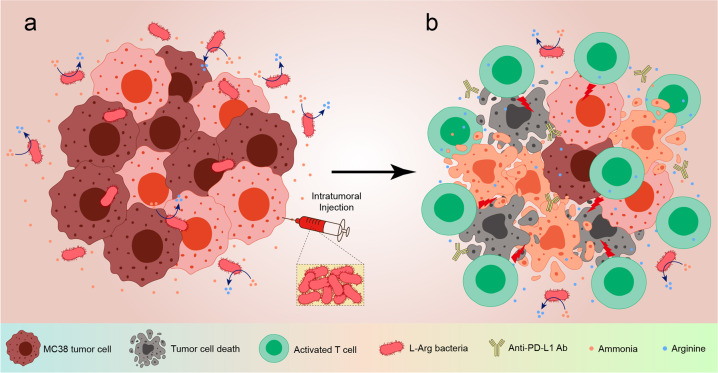
Intratumoral injection of L-Arg bacteria (**a**) shows synergistic anti-tumor effect with anti-PD-L1 antibodies which is dependent on T cell activation (**b**), causing significant tumor eradication and prolonged survival of tumor-bearing mice. Created with BioRender.com

In a recent article published in *Nature*, Roger Geiger’s research group showed that the engineered bacterial strain *Escherichia coli* Nissle 1917 (ECN) could recycle ammonia into L-arginine (Arg) in the tumor microenvironment (TME), reactivating T cells and exhibiting synergistic anti-tumor effect with anti-programmed cell death ligand 1 (PD-L1) immunotherapy in mouse MC38 tumor models (Fig. 1).^1^

Modulating TME, especially the local metabolic context, has been long proposed as a promising strategy in cancer immunotherapy. T cells are the main force against solid tumors in the latest generation of immunotherapy; however, tumor cells develop many ways to escape or repress the killing activity of tumor-reacting T cells, such as excluding T cells out of the tumor, eliciting T cell exhaustion, and competing with intratumoral T cells on the scarce nutrients including glucose, amino acids, and fatty acids. Among these substrates, Arg serves as an essential and versatile amino acid in maintaining CD3ζ expression and responsiveness of T cells.^2^ In the previous study, Roger Geiger and colleagues demonstrated that Arg could regulate several metabolic pathways such as glycolysis and oxidative phosphorylation (OXPHOS) in activated T cells, enhance T cell survival and transition to central memory-like T cells with long-term anti-tumor activity in a mouse model.^3^ Other studies also confirmed that elevating local Arg level in the TME could be a promising therapeutic strategy to improve immunotherapy.^4^

However, clinical cancer treatment with Arg remains a problem because oral administration of Arg supplementation is not practical as patients must take an impracticably high dose (150 g/75 kg/day) over the safe dose in order to achieve the expected anti-tumor effect.^1^ Although Arg can also be intratumorally injected, the observed result is not satisfactory, probably due to the rapid diffusion of Arg out of tumors.^1^ To solve the problem, it is necessary and urgent to develop alternative methods to maintain relatively high Arg concentration for long in the local TME.

Since bacteria can be detected in many types of tumors, and *E. coli* has been widely used in the delivery and release of targeted drugs in preclinical and clinical research, in the present study, Roger Geiger’s research group engineered a non-pathogenic strain of *E. coli* (ECN) to efficiently use ammonia, a metabolic waste produced by tumors, for continuous Arg synthesis and therefore sustain high local concentration of Arg in TME (termed as L-Arg bacteria). In the mice bearing subcutaneous MC38 tumors, intratumoral injection of L-Arg bacteria caused an increase in tumor-infiltrating lymphocytes (TILs) and a decrease in regulatory T cells, significantly inhibited tumor growth and prolonged mice survival, showing superior anti-tumor efficacy (Fig. 1). What’s more, L-Arg bacteria also synergistically enhanced anti-PD-L1 therapy, resulting in complete tumor eradication in 74% of MC38-bearing mice. Tumor rechallenge 90 days after complete tumor remission also confirmed the anti-tumor memory of T cells against MC38 cells in mice, which was, however, not applicable in the context of melanoma B16 cells rechallenge. Combined treatment of L-Arg bacteria and anti-PD-L1 worked well on inhibiting B16 tumor growth only in the condition that OT-I T cells were transferred after B16-OVA tumors were established. This difference may be due to the lack of available and effective anti-tumor T cell response that could be enhanced by Arg and anti-PD-L1 antibodies within tumors, and to some extent limit the interpretation and expectation of this combined therapy towards potential clinical use.

Live tumor-targeting bacteria have been proposed as an option for treating solid tumors in addition to targeted therapy and immunotherapy, and some of these engineered bacteria have even reached clinical trials.^5^ The current study has advanced our understanding about how important it is to modulate metabolic TME to support anti-tumor therapy and has broadened our research routines towards novel strategy targeting solid tumors. However, this research only shows us a tip of the iceberg, and leaves many questions to be answered and works to be done.

First, more detailed molecular mechanism by which L-Arg bacteria alone or combined with PD-L1 blockade exert anti-tumor activity needs to be fully elucidated. The outcome is attractive, but “T cell dependent” may be not enough. What signal recruits and activates these T cells? Where are they from and how come? What truly makes the therapeutic difference among different tumor types given the same treatment? Does tumor tropism or heterogeneity count? If these and other questions can be answered, maybe we can do a lot to optimize the tumor-killing effect of L-Arg bacteria combined with other therapeutic strategies including PD-L1 antibodies, other immunotherapy, and even targeted therapy, and make them effective against more cancer types.

Second, we should strictly validate the safety and efficiency of L-Arg bacteria before clinical practice. Besides, administration approach should be further improved. Intratumoral injection cannot always achieve satisfactory tumor-killing effect especially for the tumors deep inside our body, and sometimes it is dangerous with poor compliance. Although the authors in the current study also evaluate intravenous administration of L-Arg bacteria, the data are very preliminary, and the anti-tumor effect is not comparable to that of intratumoral injection. Many works remain to be done about the further assessment of its safety via intravenous administration and improvement of the overall anti-tumor outcome.

In fact, oral administration can be the most ideal way of ingesting L-Arg bacteria. As Roger Geiger and colleagues have stated at the beginning, oral administration of Arg to achieve expected anti-tumor outcome is nearly impracticable,^1^ but how about directly eating L-Arg bacteria? Is it possible to further engineer L-Arg bacteria to specifically localize into tumors after oral administration? Can it be concentrated into a piece of probiotics supplements and taken by cancer patients regularly? Perhaps this prospect needs the support of more preclinical and clinical studies, and efforts from pharmaceutical enterprises.

Taken together, this groundbreaking study provides substantial support for the use of live bacteria to modulate TME, and has the potential for clinical translation. After extensive confirmation and evaluation of its safety and efficacy in human, we believe live bacteria therapy like L-Arg bacteria may be applied in more clinical settings, not only cancer, but a variety of other human diseases.
